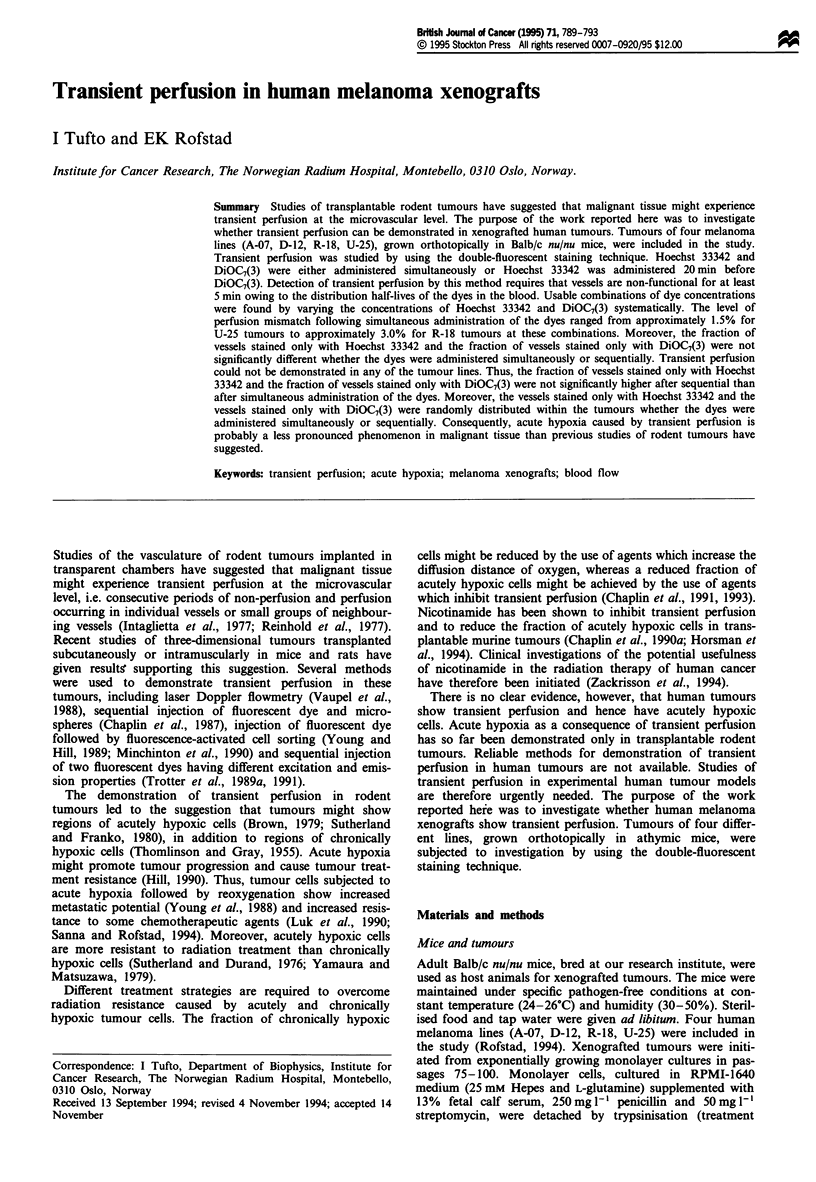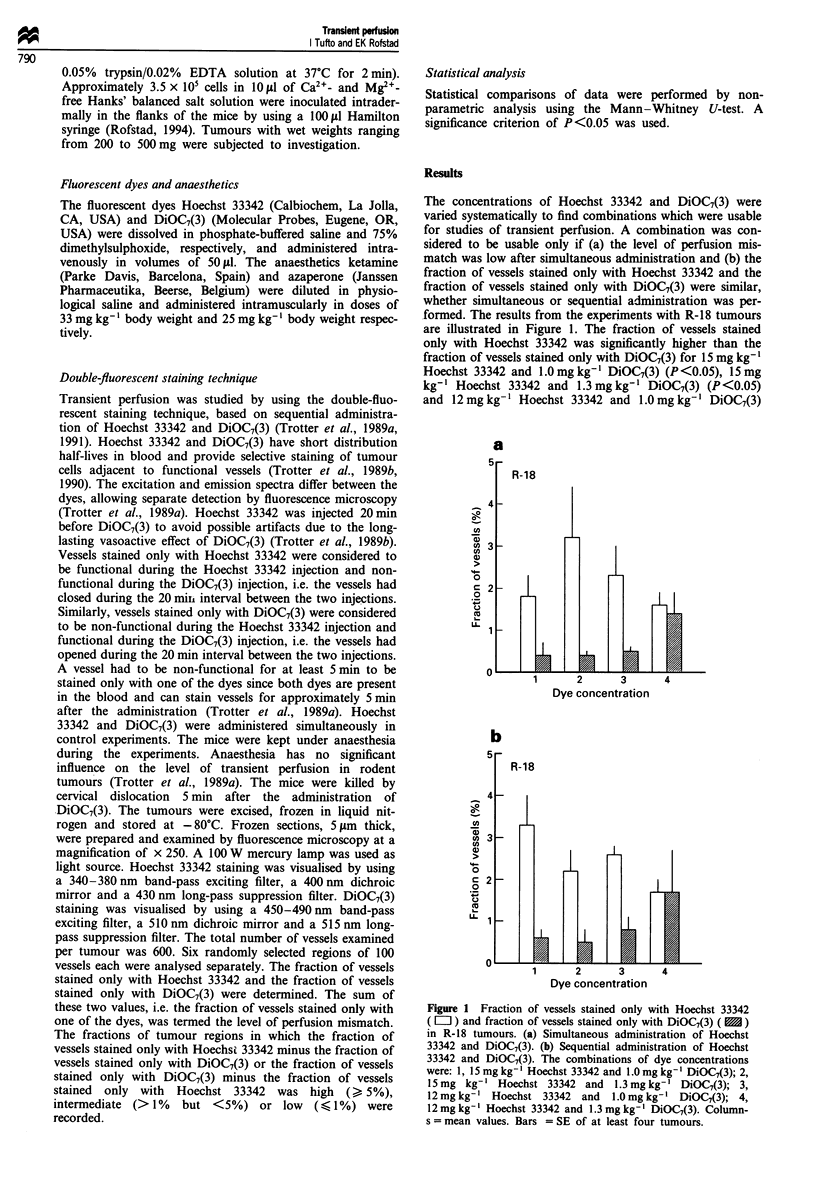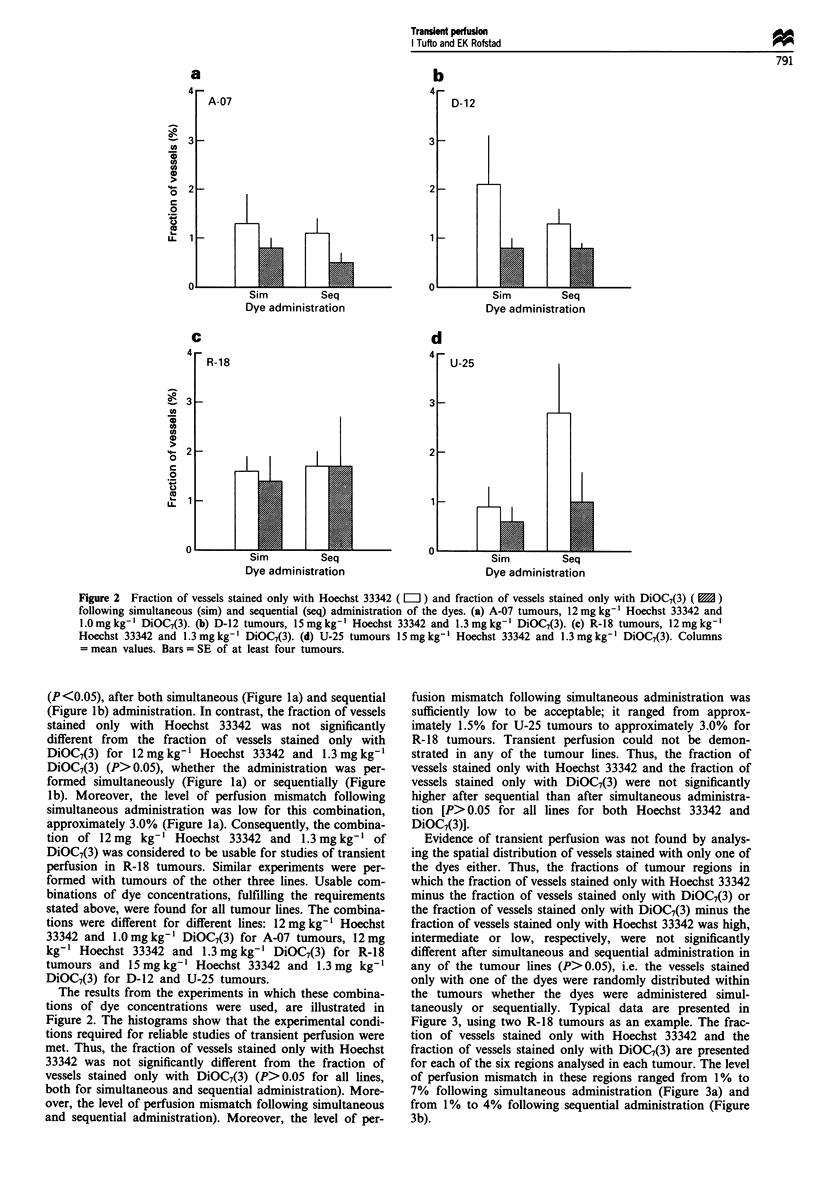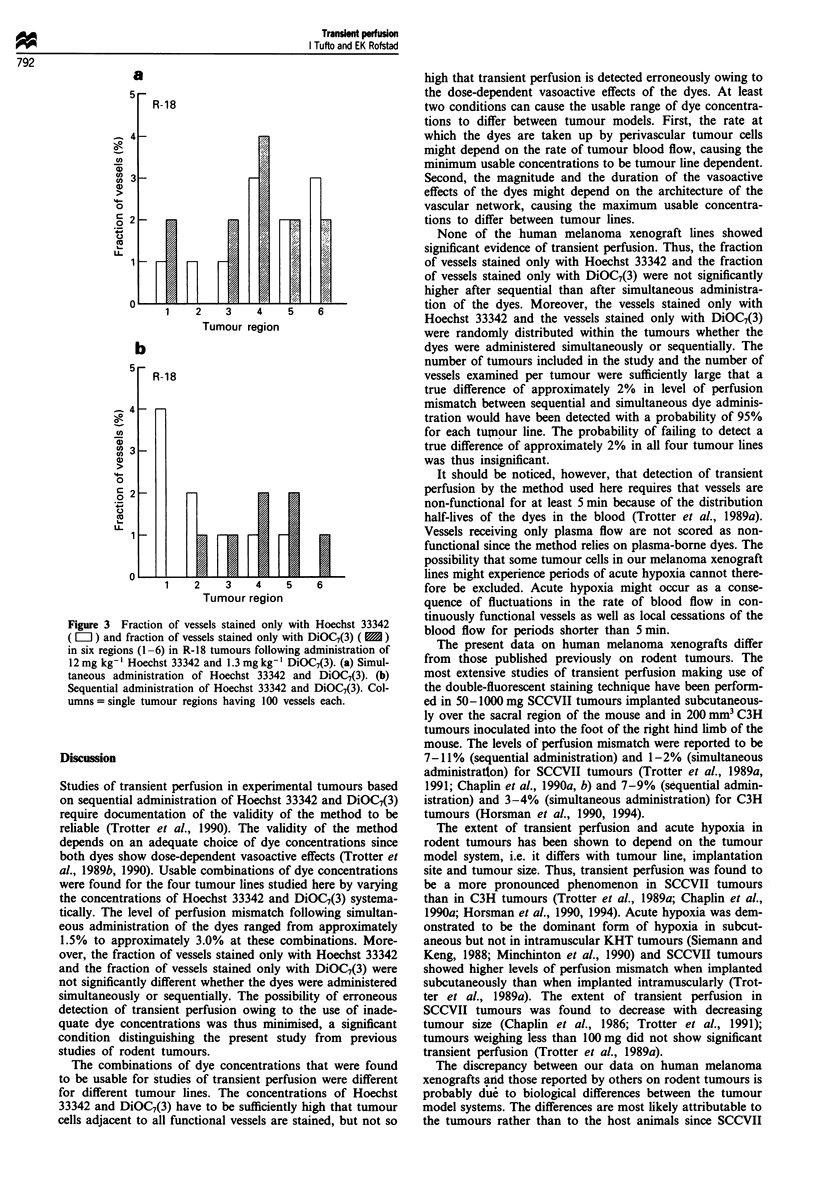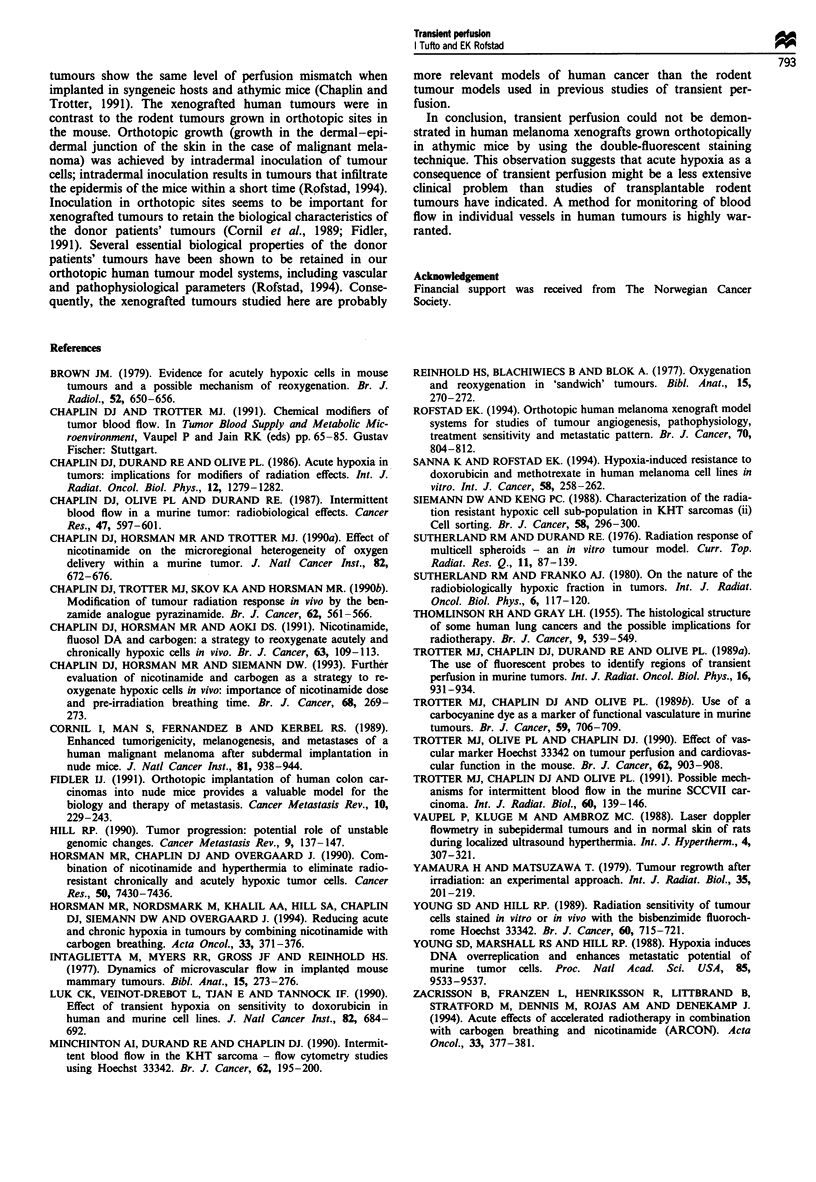# Transient perfusion in human melanoma xenografts.

**DOI:** 10.1038/bjc.1995.153

**Published:** 1995-04

**Authors:** I. Tufto, E. K. Rofstad

**Affiliations:** Institute for Cancer Research, Norwegian Radium Hospital, Montebello, Oslo.

## Abstract

Studies of transplantable rodent tumours have suggested that malignant tissue might experience transient perfusion at the microvascular level. The purpose of the work reported here was to investigate whether transient perfusion can be demonstrated in xenografted human tumours. Tumours of four melanoma lines (A-07, D-12, R-18, U-25), grown orthotopically in Balb/c nu/nu mice, were included in the study. Transient perfusion was studied by using the double-fluorescent staining technique. Hoechst 33342 and DiOC7(3) were either administered simultaneously or Hoechst 33342 was administered 20 min before DiOC7(3). Detection of transient perfusion by this method requires that vessels are non-functional for at least 5 min owing to the distribution half-lives of the dyes in the blood. Usable combinations of dye concentrations were found by varying the concentrations of Hoechst 33342 and DiOC7(3) systematically. The level of perfusion mismatch following simultaneous administration of the dyes ranged from approximately 1.5% for U-25 tumours to approximately 3.0% for R-18 tumours at these combinations. Moreover, the fraction of vessels stained only with Hoechst 33342 and the fraction of vessels stained only with DiOC7(3) were not significantly different whether the dyes were administered simultaneously or sequentially. Transient perfusion could not be demonstrated in any of the tumour lines. Thus, the fraction of vessels stained only with Hoechst 33342 and the fraction of vessels stained only with DiOC7(3) were not significantly higher after sequential than after simultaneous administration of the dyes. Moreover, the vessels stained only with Hoechst 33342 and the vessels stained only with DiOC7(3) were randomly distributed within the tumours whether the dyes were administered simultaneously or sequentially. Consequently, acute hypoxia caused by transient perfusion is probably a less pronounced phenomenon in malignant tissue than previous studies of rodent tumours have suggested.


British Journal of Cancer (1995) 71, 789-793
© 1995 Stockton Press All rights reserved 0007-0920/95 $12.00

### Transient perfusion in human melanoma xenografts

I Tufto and EK Rofstad

*Institute for Cancer Research, The Norwegian Radium Hospital, Montebello, 0310 Oslo, Norway.*

**Summary** Studies of transplantable rodent tumours have suggested that malignant tissue might experience transient perfusion at the microvascular level. The purpose of the work reported here was to investigate whether transient perfusion can be demonstrated in xenografted human tumours. Tumours of four melanoma lines (A-07, D-12, R-18, U-25), grown orthotopically in Balb/c *nu/nu* mice, were included in the study. Transient perfusion was studied by using the double-fluorescent staining technique. Hoechst 33342 and $DiOC_7(3)$ were either administered simultaneously or Hoechst 33342 was administered 20 min before $DiOC_7(3)$. Detection of transient perfusion by this method requires that vessels are non-functional for at least 5 min owing to the distribution half-lives of the dyes in the blood. Usable combinations of dye concentrations were found by varying the concentrations of Hoechst 33342 and $DiOC_7(3)$ systematically. The level of perfusion mismatch following simultaneous administration of the dyes ranged from approximately 1.5% for U-25 tumours to approximately 3.0% for R-18 tumours at these combinations. Moreover, the fraction of vessels stained only with Hoechst 33342 and the fraction of vessels stained only with $DiOC_7(3)$ were not significantly different whether the dyes were administered simultaneously or sequentially. Transient perfusion could not be demonstrated in any of the tumour lines. Thus, the fraction of vessels stained only with Hoechst 33342 and the fraction of vessels stained only with $DiOC_7(3)$ were not significantly higher after sequential than after simultaneous administration of the dyes. Moreover, the vessels stained only with Hoechst 33342 and the vessels stained only with $DiOC_7(3)$ were randomly distributed within the tumours whether the dyes were administered simultaneously or sequentially. Consequently, acute hypoxia caused by transient perfusion is probably a less pronounced phenomenon in malignant tissue than previous studies of rodent tumours have suggested.

**Keywords:** transient perfusion; acute hypoxia; melanoma xenografts; blood flow

Studies of the vasculature of rodent tumours implanted in transparent chambers have suggested that malignant tissue might experience transient perfusion at the microvascular level, i.e. consecutive periods of non-perfusion and perfusion occurring in individual vessels or small groups of neighbouring vessels (Intaglietta *et al.*, 1977; Reinhold *et al.*, 1977). Recent studies of three-dimensional tumours transplanted subcutaneously or intramuscularly in mice and rats have given results supporting this suggestion. Several methods were used to demonstrate transient perfusion in these tumours, including laser Doppler flowmetry (Vaupel *et al.*, 1988), sequential injection of fluorescent dye and microspheres (Chaplin *et al.*, 1987), injection of fluorescent dye followed by fluorescence-activated cell sorting (Young and Hill, 1989; Minchinton *et al.*, 1990) and sequential injection of two fluorescent dyes having different excitation and emission properties (Trotter *et al.*, 1989*a*, 1991).

The demonstration of transient perfusion in rodent tumours led to the suggestion that tumours might show regions of acutely hypoxic cells (Brown, 1979; Sutherland and Franko, 1980), in addition to regions of chronically hypoxic cells (Thomlinson and Gray, 1955). Acute hypoxia might promote tumour progression and cause tumour treatment resistance (Hill, 1990). Thus, tumour cells subjected to acute hypoxia followed by reoxygenation show increased metastatic potential (Young *et al.*, 1988) and increased resistance to some chemotherapeutic agents (Luk *et al.*, 1990; Sanna and Rofstad, 1994). Moreover, acutely hypoxic cells are more resistant to radiation treatment than chronically hypoxic cells (Sutherland and Durand, 1976; Yamaura and Matsuzawa, 1979).

Different treatment strategies are required to overcome radiation resistance caused by acutely and chronically hypoxic tumour cells. The fraction of chronically hypoxic cells might be reduced by the use of agents which increase the diffusion distance of oxygen, whereas a reduced fraction of acutely hypoxic cells might be achieved by the use of agents which inhibit transient perfusion (Chaplin *et al.*, 1991, 1993). Nicotinamide has been shown to inhibit transient perfusion and to reduce the fraction of acutely hypoxic cells in transplantable murine tumours (Chaplin *et al.*, 1990*a*; Horsman *et al.*, 1994). Clinical investigations of the potential usefulness of nicotinamide in the radiation therapy of human cancer have therefore been initiated (Zackrisson *et al.*, 1994).

There is no clear evidence, however, that human tumours show transient perfusion and hence have acutely hypoxic cells. Acute hypoxia as a consequence of transient perfusion has so far been demonstrated only in transplantable rodent tumours. Reliable methods for demonstration of transient perfusion in human tumours are not available. Studies of transient perfusion in experimental human tumour models are therefore urgently needed. The purpose of the work reported here was to investigate whether human melanoma xenografts show transient perfusion. Tumours of four different lines, grown orthotopically in athymic mice, were subjected to investigation by using the double-fluorescent staining technique.

### Materials and methods

### *Mice and tumours*

Adult Balb/c *nu/nu* mice, bred at our research institute, were used as host animals for xenografted tumours. The mice were maintained under specific pathogen-free conditions at constant temperature (24-26°C) and humidity (30-50%). Sterilised food and tap water were given *ad libitum*. Four human melanoma lines (A-07, D-12, R-18, U-25) were included in the study (Rofstad, 1994). Xenografted tumours were initiated from exponentially growing monolayer cultures in passages 75-100. Monolayer cells, cultured in RPMI-1640 medium (25 mM Hepes and L-glutamine) supplemented with 13% fetal calf serum, $250 \text{ mg } l^{-1}$ penicillin and $50 \text{ mg } l^{-1}$ streptomycin, were detached by trypsinisation (treatment

Correspondence: I Tufto, Department of Biophysics, Institute for Cancer Research, The Norwegian Radium Hospital, Montebello, 0310 Oslo, Norway
Received 13 September 1994; revised 4 November 1994; accepted 14 November

0.05% trypsin/0.02% EDTA solution at 37°C for 2 min). Approximately $3.5 \times 10^5$ cells in 10 µl of $Ca^{2+}$- and $Mg^{2+}$-free Hanks' balanced salt solution were inoculated intradermally in the flanks of the mice by using a 100 µl Hamilton syringe (Rofstad, 1994). Tumours with wet weights ranging from 200 to 500 mg were subjected to investigation.

*Fluorescent dyes and anaesthetics*

The fluorescent dyes Hoechst 33342 (Calbiochem, La Jolla, CA, USA) and $DiOC_7(3)$ (Molecular Probes, Eugene, OR, USA) were dissolved in phosphate-buffered saline and 75% dimethylsulphoxide, respectively, and administered intravenously in volumes of 50 µl. The anaesthetics ketamine (Parke Davis, Barcelona, Spain) and azaperone (Janssen Pharmaceutika, Beerse, Belgium) were diluted in physiological saline and administered intramuscularly in doses of 33 mg $kg^{-1}$ body weight and 25 mg $kg^{-1}$ body weight respectively.

*Double-fluorescent staining technique*

Transient perfusion was studied by using the double-fluorescent staining technique, based on sequential administration of Hoechst 33342 and $DiOC_7(3)$ (Trotter *et al.*, 1989*a*, 1991). Hoechst 33342 and $DiOC_7(3)$ have short distribution half-lives in blood and provide selective staining of tumour cells adjacent to functional vessels (Trotter *et al.*, 1989*b*, 1990). The excitation and emission spectra differ between the dyes, allowing separate detection by fluorescence microscopy (Trotter *et al.*, 1989*a*). Hoechst 33342 was injected 20 min before $DiOC_7(3)$ to avoid possible artifacts due to the long-lasting vasoactive effect of $DiOC_7(3)$ (Trotter *et al.*, 1989*b*). Vessels stained only with Hoechst 33342 were considered to be functional during the Hoechst 33342 injection and non-functional during the $DiOC_7(3)$ injection, i.e. the vessels had closed during the 20 min interval between the two injections. Similarly, vessels stained only with $DiOC_7(3)$ were considered to be non-functional during the Hoechst 33342 injection and functional during the $DiOC_7(3)$ injection, i.e. the vessels had opened during the 20 min interval between the two injections. A vessel had to be non-functional for at least 5 min to be stained only with one of the dyes since both dyes are present in the blood and can stain vessels for approximately 5 min after the administration (Trotter *et al.*, 1989*a*). Hoechst 33342 and $DiOC_7(3)$ were administered simultaneously in control experiments. The mice were kept under anaesthesia during the experiments. Anaesthesia has no significant influence on the level of transient perfusion in rodent tumours (Trotter *et al.*, 1989*a*). The mice were killed by cervical dislocation 5 min after the administration of $DiOC_7(3)$. The tumours were excised, frozen in liquid nitrogen and stored at −80°C. Frozen sections, 5 µm thick, were prepared and examined by fluorescence microscopy at a magnification of × 250. A 100 W mercury lamp was used as light source. Hoechst 33342 staining was visualised by using a 340–380 nm band-pass exciting filter, a 400 nm dichroic mirror and a 430 nm long-pass suppression filter. $DiOC_7(3)$ staining was visualised by using a 450–490 nm band-pass exciting filter, a 510 nm dichroic mirror and a 515 nm long-pass suppression filter. The total number of vessels examined per tumour was 600. Six randomly selected regions of 100 vessels each were analysed separately. The fraction of vessels stained only with Hoechst 33342 and the fraction of vessels stained only with $DiOC_7(3)$ were determined. The sum of these two values, i.e. the fraction of vessels stained only with one of the dyes, was termed the level of perfusion mismatch. The fractions of tumour regions in which the fraction of vessels stained only with Hoechst 33342 minus the fraction of vessels stained only with $DiOC_7(3)$ or the fraction of vessels stained only with $DiOC_7(3)$ minus the fraction of vessels stained only with Hoechst 33342 was high ($\geqslant$5%), intermediate (>1% but <5%) or low ($\leqslant$1%) were recorded.

*Statistical analysis*

Statistical comparisons of data were performed by non-parametric analysis using the Mann–Whitney *U*-test. A significance criterion of $P<0.05$ was used.

### Results

The concentrations of Hoechst 33342 and $DiOC_7(3)$ were varied systematically to find combinations which were usable for studies of transient perfusion. A combination was considered to be usable only if (a) the level of perfusion mismatch was low after simultaneous administration and (b) the fraction of vessels stained only with Hoechst 33342 and the fraction of vessels stained only with $DiOC_7(3)$ were similar, whether simultaneous or sequential administration was performed. The results from the experiments with R-18 tumours are illustrated in Figure 1. The fraction of vessels stained only with Hoechst 33342 was significantly higher than the fraction of vessels stained only with $DiOC_7(3)$ for 15 mg $kg^{-1}$ Hoechst 33342 and 1.0 mg $kg^{-1}$ $DiOC_7(3)$ ($P<0.05$), 15 mg $kg^{-1}$ Hoechst 33342 and 1.3 mg $kg^{-1}$ $DiOC_7(3)$ ($P<0.05$) and 12 mg $kg^{-1}$ Hoechst 33342 and 1.0 mg $kg^{-1}$ $DiOC_7(3)$

**Figure 1** Fraction of vessels stained only with Hoechst 33342 ( ☐ ) and fraction of vessels stained only with $DiOC_7(3)$ ( ▨ ) in R-18 tumours. (**a**) Simultaneous administration of Hoechst 33342 and $DiOC_7(3)$. (**b**) Sequential administration of Hoechst 33342 and $DiOC_7(3)$. The combinations of dye concentrations were: 1, 15 mg $kg^{-1}$ Hoechst 33342 and 1.0 mg $kg^{-1}$ $DiOC_7(3)$; 2, 15 mg $kg^{-1}$ Hoechst 33342 and 1.3 mg $kg^{-1}$ $DiOC_7(3)$; 3, 12 mg $kg^{-1}$ Hoechst 33342 and 1.0 mg $kg^{-1}$ $DiOC_7(3)$; 4, 12 mg $kg^{-1}$ Hoechst 33342 and 1.3 mg $kg^{-1}$ $DiOC_7(3)$. Columns = mean values. Bars = SE of at least four tumours.

**Figure 2** Fraction of vessels stained only with Hoechst 33342 ( □ ) and fraction of vessels stained only with $DiOC_7(3)$ ( ▨ ) following simultaneous (sim) and sequential (seq) administration of the dyes. (**a**) A-07 tumours, 12 mg $kg^{-1}$ Hoechst 33342 and 1.0 mg $kg^{-1}$ $DiOC_7(3)$. (**b**) D-12 tumours, 15 mg $kg^{-1}$ Hoechst 33342 and 1.3 mg $kg^{-1}$ $DiOC_7(3)$. (**c**) R-18 tumours, 12 mg $kg^{-1}$ Hoechst 33342 and 1.3 mg $kg^{-1}$ $DiOC_7(3)$. (**d**) U-25 tumours 15 mg $kg^{-1}$ Hoechst 33342 and 1.3 mg $kg^{-1}$ $DiOC_7(3)$. Columns = mean values. Bars = SE of at least four tumours.

($P<0.05$), after both simultaneous (Figure 1a) and sequential (Figure 1b) administration. In contrast, the fraction of vessels stained only with Hoechst 33342 was not significantly different from the fraction of vessels stained only with $DiOC_7(3)$ for 12 mg $kg^{-1}$ Hoechst 33342 and 1.3 mg $kg^{-1}$ $DiOC_7(3)$ ($P>0.05$), whether the administration was performed simultaneously (Figure 1a) or sequentially (Figure 1b). Moreover, the level of perfusion mismatch following simultaneous administration was low for this combination, approximately 3.0% (Figure 1a). Consequently, the combination of 12 mg $kg^{-1}$ Hoechst 33342 and 1.3 mg $kg^{-1}$ of $DiOC_7(3)$ was considered to be usable for studies of transient perfusion in R-18 tumours. Similar experiments were performed with tumours of the other three lines. Usable combinations of dye concentrations, fulfilling the requirements stated above, were found for all tumour lines. The combinations were different for different lines: 12 mg $kg^{-1}$ Hoechst 33342 and 1.0 mg $kg^{-1}$ $DiOC_7(3)$ for A-07 tumours, 12 mg $kg^{-1}$ Hoechst 33342 and 1.3 mg $kg^{-1}$ $DiOC_7(3)$ for R-18 tumours and 15 mg $kg^{-1}$ Hoechst 33342 and 1.3 mg $kg^{-1}$ $DiOC_7(3)$ for D-12 and U-25 tumours.

The results from the experiments in which these combinations of dye concentrations were used, are illustrated in Figure 2. The histograms show that the experimental conditions required for reliable studies of transient perfusion were met. Thus, the fraction of vessels stained only with Hoechst 33342 was not significantly different from the fraction of vessels stained only with $DiOC_7(3)$ ($P>0.05$ for all lines, both for simultaneous and sequential administration). Moreover, the level of perfusion mismatch following simultaneous and sequential administration). Moreover, the level of perfusion mismatch following simultaneous administration was sufficiently low to be acceptable; it ranged from approximately 1.5% for U-25 tumours to approximately 3.0% for R-18 tumours. Transient perfusion could not be demonstrated in any of the tumour lines. Thus, the fraction of vessels stained only with Hoechst 33342 and the fraction of vessels stained only with $DiOC_7(3)$ were not significantly higher after sequential than after simultaneous administration [$P>0.05$ for all lines for both Hoechst 33342 and $DiOC_7(3)$].

Evidence of transient perfusion was not found by analysing the spatial distribution of vessels stained with only one of the dyes either. Thus, the fractions of tumour regions in which the fraction of vessels stained only with Hoechst 33342 minus the fraction of vessels stained only with $DiOC_7(3)$ or the fraction of vessels stained only with $DiOC_7(3)$ minus the fraction of vessels stained only with Hoechst 33342 was high, intermediate or low, respectively, were not significantly different after simultaneous and sequential administration in any of the tumour lines ($P>0.05$), i.e. the vessels stained only with one of the dyes were randomly distributed within the tumours whether the dyes were administered simultaneously or sequentially. Typical data are presented in Figure 3, using two R-18 tumours as an example. The fraction of vessels stained only with Hoechst 33342 and the fraction of vessels stained only with $DiOC_7(3)$ are presented for each of the six regions analysed in each tumour. The level of perfusion mismatch in these regions ranged from 1% to 7% following simultaneous administration (Figure 3a) and from 1% to 4% following sequential administration (Figure 3b).

**Figure 3** Fraction of vessels stained only with Hoechst 33342 (□) and fraction of vessels stained only with $DiOC_7(3)$ (▨) in six regions (1–6) in R-18 tumours following administration of 12 mg $kg^{-1}$ Hoechst 33342 and 1.3 mg $kg^{-1}$ $DiOC_7(3)$. (**a**) Simultaneous administration of Hoechst 33342 and $DiOC_7(3)$. (**b**) Sequential administration of Hoechst 33342 and $DiOC_7(3)$. Columns = single tumour regions having 100 vessels each.

### Discussion

Studies of transient perfusion in experimental tumours based on sequential administration of Hoechst 33342 and $DiOC_7(3)$ require documentation of the validity of the method to be reliable (Trotter *et al.*, 1990). The validity of the method depends on an adequate choice of dye concentrations since both dyes show dose-dependent vasoactive effects (Trotter *et al.*, 1989*b*, 1990). Usable combinations of dye concentrations were found for the four tumour lines studied here by varying the concentrations of Hoechst 33342 and $DiOC_7(3)$ systematically. The level of perfusion mismatch following simultaneous administration of the dyes ranged from approximately 1.5% to approximately 3.0% at these combinations. Moreover, the fraction of vessels stained only with Hoechst 33342 and the fraction of vessels stained only with $DiOC_7(3)$ were not significantly different whether the dyes were administered simultaneously or sequentially. The possibility of erroneous detection of transient perfusion owing to the use of inadequate dye concentrations was thus minimised, a significant condition distinguishing the present study from previous studies of rodent tumours.

The combinations of dye concentrations that were found to be usable for studies of transient perfusion were different for different tumour lines. The concentrations of Hoechst 33342 and $DiOC_7(3)$ have to be sufficiently high that tumour cells adjacent to all functional vessels are stained, but not so high that transient perfusion is detected erroneously owing to the dose-dependent vasoactive effects of the dyes. At least two conditions can cause the usable range of dye concentrations to differ between tumour models. First, the rate at which the dyes are taken up by perivascular tumour cells might depend on the rate of tumour blood flow, causing the minimum usable concentrations to be tumour line dependent. Second, the magnitude and the duration of the vasoactive effects of the dyes might depend on the architecture of the vascular network, causing the maximum usable concentrations to differ between tumour lines.

None of the human melanoma xenograft lines showed significant evidence of transient perfusion. Thus, the fraction of vessels stained only with Hoechst 33342 and the fraction of vessels stained only with $DiOC_7(3)$ were not significantly higher after sequential than after simultaneous administration of the dyes. Moreover, the vessels stained only with Hoechst 33342 and the vessels stained only with $DiOC_7(3)$ were randomly distributed within the tumours whether the dyes were administered simultaneously or sequentially. The number of tumours included in the study and the number of vessels examined per tumour were sufficiently large that a true difference of approximately 2% in level of perfusion mismatch between sequential and simultaneous dye administration would have been detected with a probability of 95% for each tumour line. The probability of failing to detect a true difference of approximately 2% in all four tumour lines was thus insignificant.

It should be noticed, however, that detection of transient perfusion by the method used here requires that vessels are non-functional for at least 5 min because of the distribution half-lives of the dyes in the blood (Trotter *et al.*, 1989*a*). Vessels receiving only plasma flow are not scored as non-functional since the method relies on plasma-borne dyes. The possibility that some tumour cells in our melanoma xenograft lines might experience periods of acute hypoxia cannot therefore be excluded. Acute hypoxia might occur as a consequence of fluctuations in the rate of blood flow in continuously functional vessels as well as local cessations of the blood flow for periods shorter than 5 min.

The present data on human melanoma xenografts differ from those published previously on rodent tumours. The most extensive studies of transient perfusion making use of the double-fluorescent staining technique have been performed in 50–1000 mg SCCVII tumours implanted subcutaneously over the sacral region of the mouse and in 200 $mm^3$ C3H tumours inoculated into the foot of the right hind limb of the mouse. The levels of perfusion mismatch were reported to be 7–11% (sequential administration) and 1–2% (simultaneous administration) for SCCVII tumours (Trotter *et al.*, 1989*a*, 1991; Chaplin *et al.*, 1990*a*, *b*) and 7–9% (sequential administration) and 3–4% (simultaneous administration) for C3H tumours (Horsman *et al.*, 1990, 1994).

The extent of transient perfusion and acute hypoxia in rodent tumours has been shown to depend on the tumour model system, i.e. it differs with tumour line, implantation site and tumour size. Thus, transient perfusion was found to be a more pronounced phenomenon in SCCVII tumours than in C3H tumours (Trotter *et al.*, 1989*a*; Chaplin *et al.*, 1990*a*; Horsman *et al.*, 1990, 1994). Acute hypoxia was demonstrated to be the dominant form of hypoxia in subcutaneous but not in intramuscular KHT tumours (Siemann and Keng, 1988; Minchinton *et al.*, 1990) and SCCVII tumours showed higher levels of perfusion mismatch when implanted subcutaneously than when implanted intramuscularly (Trotter *et al.*, 1989*a*). The extent of transient perfusion in SCCVII tumours was found to decrease with decreasing tumour size (Chaplin *et al.*, 1986; Trotter *et al.*, 1991); tumours weighing less than 100 mg did not show significant transient perfusion (Trotter *et al.*, 1989*a*).

The discrepancy between our data on human melanoma xenografts and those reported by others on rodent tumours is probably due to biological differences between the tumour model systems. The differences are most likely attributable to the tumours rather than to the host animals since SCCVII

tumours show the same level of perfusion mismatch when implanted in syngeneic hosts and athymic mice (Chaplin and Trotter, 1991). The xenografted human tumours were in contrast to the rodent tumours grown in orthotopic sites in the mouse. Orthotopic growth (growth in the dermal–epidermal junction of the skin in the case of malignant melanoma) was achieved by intradermal inoculation of tumour cells; intradermal inoculation results in tumours that infiltrate the epidermis of the mice within a short time (Rofstad, 1994). Inoculation in orthotopic sites seems to be important for xenografted tumours to retain the biological characteristics of the donor patients' tumours (Cornil *et al.*, 1989; Fidler, 1991). Several essential biological properties of the donor patients' tumours have been shown to be retained in our orthotopic human tumour model systems, including vascular and pathophysiological parameters (Rofstad, 1994). Consequently, the xenografted tumours studied here are probably more relevant models of human cancer than the rodent tumour models used in previous studies of transient perfusion.

In conclusion, transient perfusion could not be demonstrated in human melanoma xenografts grown orthotopically in athymic mice by using the double-fluorescent staining technique. This observation suggests that acute hypoxia as a consequence of transient perfusion might be a less extensive clinical problem than studies of transplantable rodent tumours have indicated. A method for monitoring of blood flow in individual vessels in human tumours is highly warranted.

**Acknowledgement**

Financial support was received from The Norwegian Cancer Society.

**References**

BROWN JM. (1979). Evidence for acutely hypoxic cells in mouse tumours and a possible mechanism of reoxygenation. *Br. J. Radiol.*, **52,** 650–656.

CHAPLIN DJ AND TROTTER MJ. (1991). Chemical modifiers of tumor blood flow. In *Tumor Blood Supply and Metabolic Microenvironment*, Vaupel P and Jain RK (eds) pp. 65–85. Gustav Fischer: Stuttgart.

CHAPLIN DJ, DURAND RE AND OLIVE PL. (1986). Acute hypoxia in tumors: implications for modifiers of radiation effects. *Int. J. Radiat. Oncol. Biol. Phys.*, **12,** 1279–1282.

CHAPLIN DJ, OLIVE PL AND DURAND RE. (1987). Intermittent blood flow in a murine tumor: radiobiological effects. *Cancer Res.*, **47,** 597–601.

CHAPLIN DJ, HORSMAN MR AND TROTTER MJ. (1990*a*). Effect of nicotinamide on the microregional heterogeneity of oxygen delivery within a murine tumor. *J. Natl Cancer Inst.*, **82,** 672–676.

CHAPLIN DJ, TROTTER MJ, SKOV KA AND HORSMAN MR. (1990*b*). Modification of tumour radiation response *in vivo* by the benzamide analogue pyrazinamide. *Br. J. Cancer*, **62,** 561–566.

CHAPLIN DJ, HORSMAN MR AND AOKI DS. (1991). Nicotinamide, fluosol DA and carbogen: a strategy to reoxygenate acutely and chronically hypoxic cells *in vivo*. *Br. J. Cancer*, **63,** 109–113.

CHAPLIN DJ, HORSMAN MR AND SIEMANN DW. (1993). Further evaluation of nicotinamide and carbogen as a strategy to reoxygenate hypoxic cells *in vivo*: importance of nicotinamide dose and pre-irradiation breathing time. *Br. J. Cancer*, **68,** 269–273.

CORNIL I, MAN S, FERNANDEZ B AND KERBEL RS. (1989). Enhanced tumorigenicity, melanogenesis, and metastases of a human malignant melanoma after subdermal implantation in nude mice. *J. Natl Cancer Inst.*, **81,** 938–944.

FIDLER IJ. (1991). Orthotopic implantation of human colon carcinomas into nude mice provides a valuable model for the biology and therapy of metastasis. *Cancer Metastasis Rev.*, **10,** 229–243.

HILL RP. (1990). Tumor progression: potential role of unstable genomic changes. *Cancer Metastasis Rev.*, **9,** 137–147.

HORSMAN MR, CHAPLIN DJ AND OVERGAARD J. (1990). Combination of nicotinamide and hyperthermia to eliminate radioresistant chronically and acutely hypoxic tumor cells. *Cancer Res.*, **50,** 7430–7436.

HORSMAN MR, NORDSMARK M, KHALIL AA, HILL SA, CHAPLIN DJ, SIEMANN DW AND OVERGAARD J. (1994). Reducing acute and chronic hypoxia in tumours by combining nicotinamide with carbogen breathing. *Acta Oncol.*, **33,** 371–376.

INTAGLIETTA M, MYERS RR, GROSS JF AND REINHOLD HS. (1977). Dynamics of microvascular flow in implanted mouse mammary tumours. *Bibl. Anat.*, **15,** 273–276.

LUK CK, VEINOT-DREBOT L, TJAN E AND TANNOCK IF. (1990). Effect of transient hypoxia on sensitivity to doxorubicin in human and murine cell lines. *J. Natl Cancer Inst.*, **82,** 684–692.

MINCHINTON AI, DURAND RE AND CHAPLIN DJ. (1990). Intermittent blood flow in the KHT sarcoma – flow cytometry studies using Hoechst 33342. *Br. J. Cancer*, **62,** 195–200.

REINHOLD HS, BLACHIWIECS B AND BLOK A. (1977). Oxygenation and reoxygenation in 'sandwich' tumours. *Bibl. Anat.*, **15,** 270–272.

ROFSTAD EK. (1994). Orthotopic human melanoma xenograft model systems for studies of tumour angiogenesis, pathophysiology, treatment sensitivity and metastatic pattern. *Br. J. Cancer*, **70,** 804–812.

SANNA K AND ROFSTAD EK. (1994). Hypoxia-induced resistance to doxorubicin and methotrexate in human melanoma cell lines *in vitro*. *Int. J. Cancer*, **58,** 258–262.

SIEMANN DW AND KENG PC. (1988). Characterization of the radiation resistant hypoxic cell sub-population in KHT sarcomas (ii) Cell sorting. *Br. J. Cancer*, **58,** 296–300.

SUTHERLAND RM AND DURAND RE. (1976). Radiation response of multicell spheroids – an *in vitro* tumour model. *Curr. Top. Radiat. Res. Q.*, **11,** 87–139.

SUTHERLAND RM AND FRANKO AJ. (1980). On the nature of the radiobiologically hypoxic fraction in tumors. *Int. J. Radiat. Oncol. Biol. Phys.*, **6,** 117–120.

THOMLINSON RH AND GRAY LH. (1955). The histological structure of some human lung cancers and the possible implications for radiotherapy. *Br. J. Cancer*, **9,** 539–549.

TROTTER MJ, CHAPLIN DJ, DURAND RE AND OLIVE PL. (1989*a*). The use of fluorescent probes to identify regions of transient perfusion in murine tumors. *Int. J. Radiat. Oncol. Biol. Phys.*, **16,** 931–934.

TROTTER MJ, CHAPLIN DJ AND OLIVE PL. (1989*b*). Use of a carbocyanine dye as a marker of functional vasculature in murine tumours. *Br. J. Cancer*, **59,** 706–709.

TROTTER MJ, OLIVE PL AND CHAPLIN DJ. (1990). Effect of vascular marker Hoechst 33342 on tumour perfusion and cardiovascular function in the mouse. *Br. J. Cancer*, **62,** 903–908.

TROTTER MJ, CHAPLIN DJ AND OLIVE PL. (1991). Possible mechanisms for intermittent blood flow in the murine SCCVII carcinoma. *Int. J. Radiat. Biol.*, **60,** 139–146.

VAUPEL P, KLUGE M AND AMBROZ MC. (1988). Laser doppler flowmetry in subepidermal tumours and in normal skin of rats during localized ultrasound hyperthermia. *Int. J. Hypertherm.*, **4,** 307–321.

YAMAURA H AND MATSUZAWA T. (1979). Tumour regrowth after irradiation: an experimental approach. *Int. J. Radiat. Biol.*, **35,** 201–219.

YOUNG SD AND HILL RP. (1989). Radiation sensitivity of tumour cells stained *in vitro* or *in vivo* with the bisbenzimide fluorochrome Hoechst 33342. *Br. J. Cancer*, **60,** 715–721.

YOUNG SD, MARSHALL RS AND HILL RP. (1988). Hypoxia induces DNA overreplication and enhances metastatic potential of murine tumor cells. *Proc. Natl Acad. Sci. USA*, **85,** 9533–9537.

ZACRISSON B, FRANZEN L, HENRIKSSON R, LITTBRAND B, STRATFORD M, DENNIS M, ROJAS AM AND DENEKAMP J. (1994). Acute effects of accelerated radiotherapy in combination with carbogen breathing and nicotinamide (ARCON). *Acta Oncol.*, **33,** 377–381.